# Promoter Motifs in NCLDVs: An Evolutionary Perspective

**DOI:** 10.3390/v9010016

**Published:** 2017-01-20

**Authors:** Graziele Pereira Oliveira, Ana Cláudia dos Santos Pereira Andrade, Rodrigo Araújo Lima Rodrigues, Thalita Souza Arantes, Paulo Victor Miranda Boratto, Ludmila Karen dos Santos Silva, Fábio Pio Dornas, Giliane de Souza Trindade, Betânia Paiva Drumond, Bernard La Scola, Erna Geessien Kroon, Jônatas Santos Abrahão

**Affiliations:** 1 Laboratório de Vírus, Departamento de Microbiologia, Instituto de Ciências Biológicas, Universidade Federal de Minas Gerais, Belo Horizonte 31270-901, Minas Gerais, Brazil; graziufmg@yahoo.com.br (G.P.O.); ana.andrade2008@hotmail.com (A.C.d.S.P.A.); rodriguesral07@gmail.com (R.A.L.R.); tsarantes@gmail.com (T.S.A.); pvboratto@gmail.com (P.V.M.B.); ludmilakaren@gmail.com (L.K.d.S.S.); fabiopiod154@gmail.com (F.P.D.); gitrindade@yahoo.com.br (G.d.S.T.); betaniadrumond@gmail.com (B.P.D.); ernagkroon@gmail.com (E.G.K.); 2 Unité de Recherche sur les Maladies Infectieuses et Tropicales Emergentes (URMITE) UM63 CNRS 7278 IRD 198 INSERM U1095, Aix-Marseille Université., 27 Boulevard Jean Moulin, Faculté de Médecine, 13385 Marseille Cedex 05, France; bernard.la-scola@univ-amu.fr

**Keywords:** megavirales, NCLDV, giant viruses, promoter, transcription, evolution, MEGA-box

## Abstract

For many years, gene expression in the three cellular domains has been studied in an attempt to discover sequences associated with the regulation of the transcription process. Some specific transcriptional features were described in viruses, although few studies have been devoted to understanding the evolutionary aspects related to the spread of promoter motifs through related viral families. The discovery of giant viruses and the proposition of the new viral order Megavirales that comprise a monophyletic group, named nucleo-cytoplasmic large DNA viruses (NCLDV), raised new questions in the field. Some putative promoter sequences have already been described for some NCLDV members, bringing new insights into the evolutionary history of these complex microorganisms. In this review, we summarize the main aspects of the transcription regulation process in the three domains of life, followed by a systematic description of what is currently known about promoter regions in several NCLDVs. We also discuss how the analysis of the promoter sequences could bring new ideas about the giant viruses’ evolution. Finally, considering a possible common ancestor for the NCLDV group, we discussed possible promoters’ evolutionary scenarios and propose the term “MEGA-box” to designate an ancestor promoter motif (‘TATATAAAATTGA’) that could be evolved gradually by nucleotides’ gain and loss and point mutations.

## 1. Introduction

For decades, viruses have been strictly considered intracellular parasites, filterable in membranes of 0.22 nm, composed by genomes of DNA or RNA encoding only a few proteins, being entirely dependent on the metabolic machinery of the host cell [1]. However, viruses show a large diversity of genome size and organization, capsid architecture, mechanisms of replication, and interactions with host cells. The extreme diversity of viruses suggests that they must have had multiple evolutionary origins, thus being polyphyletic [2]. In 2001, a supposedly monophyletic group named nucleo-cytoplasmic large DNA viruses (NCLDV) was proposed, composed of families *Poxviridae*, *Asfarviridae*, *Iridoviridae* and *Phycodnaviridae* [3]. This group gained notoriety two years later with the discovery of *Acanthamoeba polyphaga mimivirus* [4] and it is currently composed of the families mentioned above, as well as *Ascoviridae*, and the more recently incorporated *Mimiviridae* and *Marseilleviridae* [5]. Moreover, other recently discovered giant viruses such as pandoraviruses, faustoviruses and pithoviruses were classified as members of the NCLDV group [6,7,8,9]. This group has single features such as large genomes and a diverse gene repertoire, which encode some proteins never identified previously in viruses. Therefore, the creation of a new viral order named ‘Megavirales’, encompassing all families of the NCLDV group was proposed [5].

This proposed order comprises viruses with large double-stranded DNA (dsDNA) genomes, encoding hundreds of proteins and capable of infecting a wide-range of eukaryotic organisms. These viruses replicate completely or partly, in the cytoplasm of eukaryotic cells and some of them are able to synthesize RNA polymerases (RNA pol), helicases and transcription factors involved in the transcription initiation and elongation steps with lower dependence of the host’s transcriptional machinery [3]. The presence of a robust transcriptional apparatus in some Megavirales members, along with a quasi-autonomous glycosylation and translational machinery, especially in mimiviruses, boosted the discussion about the origin and evolution of giant viruses and their genome. Recent evolutionary reconstructions mapped about 25–50 genes encoding essential genes for the probable most recent common ancestor [10]. Concerning the origin of such giant genomes, different hypotheses have been proposed. Some authors suggest a “genome degradation hypothesis”, wherein the giant viruses are derived from a cellular ancestor through genome simplification linked to the adaptation to some host lineage [11,12]. Other authors argue in favor of a “genome expansion hypothesis”, wherein the giant viruses evolved from a smaller viral ancestor and the universal genes have been independently acquired from their eukaryotic hosts by progressive gene accretion and duplication. According to this theory, the genes of giant viruses have several origins and the origin of giant viruses is probably from a simpler ancestor [13,14].

On the other hand, the accordion-like model of evolution proposes that there is no trend of genome expansion or general tendency of genome contraction. Instead, viruses evolving by constant gene gain and loss originated from an ancestor giant virus [10]. All these theories are often contradictory and have stimulated discussion about the establishment of a fourth domain of life where the giant viruses of the proposed order Megavirales were suggested to share a common ancestral origin based on analyses of their sequences and gene repertoires and compose a new domain aside Bacteria, Archaea and Eukarya [14,15,16].

During the last years, a huge effort has been made to better understand the virus–host interaction on many levels. One of the most interesting research fields is how the viruses can explore host transcriptional machinery to express their genes. Nevertheless, it is important also to look into the transcription process of the cellular organisms. The upstream regions of eukaryotes and prokaryotes genes have been studied in different organisms in an attempt to discover sequences associated with the regulation of the transcription process. The same has been done for viruses, especially considering the proposed Megavirales order, where some putative promoter sequences have already been described. In this review, we summarize the main aspects of the transcription regulation process in the three domains of life, followed by a systematic description of current knowledge of the promoter regions of all members within Megavirales order. Finally, we discuss how the analysis of the promoter sequences found in giant viruses provides new insights into the evolutionary history of these complex and intriguing agents.

## 2. Gene Expression in Cells

In all cells, thousands of genes encoded in the DNA are transcribed into RNA and for the efficient occurrence of this process, multiple events must be triggered. In eukaryotes, the genome is coupled to histones and other proteins, forming the chromatin compact complex. Since wrapping DNA around histones blocks the access to the genetic information, decondensation of DNA is required, to allow physical access to the the gene locus and the transcription initiation machinery formation [17,18,19]. The transcription initiation machinery is formed over a region of the genome, the promoter. The promoter is typically located 40 bp upstream and downstream of the transcription start of a gene, called transcription start sites (TSS). Several transcription factors mediate the transcription machinery assembly on the promoter region. There are thousands of transcription factors involved in the transcription process, such as TFIIA, TFIIB, TFIID, TFIIE, TFIIF and TFIIH that recognize and bind the promoter region, called the core promoter, and recruit RNA polymerase (RNA pol) [20]. Eukaryotes have five types of RNA pol (I to V). RNA pol I transcribes ribosomal RNA, whereas the type II is the best characterized one and responsible for transcribing genes encoding proteins, and several noncoding RNA classes [18,21,22]. RNA pol III transcribes genes encoding short, untranslated RNAs, such as tRNAs, 5S ribosomal RNA (rRNA) and the spliceosomal U6 small nuclear RNA (snRNA) [23]. RNA pol IV and V transcribe siRNA in plants [24].

**Figure 1:**
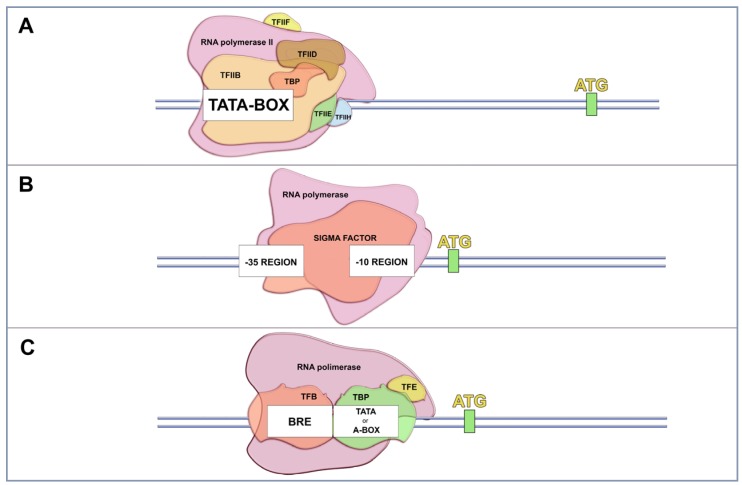
Main features in the transcription initiation machinery presented in the canonical Domains of Life. (**A**) In Eukarya, several components, called general transcription factors (represented as TFIIB, TFIID, TFIIE, TFIIF, TFIIH and TBP), are responsible for assembling over a region called the promoter, where they recruit an RNA polimerase to initiate the transcription process. A classical promoter presented in this group is the TATA-BOX region, located at the positions −25 and −30 from the initial transcription site; (**B**) In Bacteria, the sigma factor recognizes and recruits the RNA polimerase over the promoter regions. These regions are well conserved over the positions −35 and −10 upstream of the initial transcription site; (**C**) Archaea present a mixture of the transcription apparatus of the two other Domains. While the machinery itself is similar to that found in eukaryotes (the general transcription factors, a homologous TATA-BOX region and the RNA polimerase), the archaeal transcription regulators, activators and repressors are homologous to the bacterial ones.

One classical element of the core promoter is the TATA-box, which is a consensus sequence (TATAAAT) located at −25 to −30 bp upstream of the TSS. Although the TATA-box sequence is a well-known promoter core motif, it is present only in a minority of mammalian promoters. This sequence is commonly associated with tissue-specific gene transcription and high conservation within species [25,26]. Other eukariotic promoter elements are Initiator (Inr); Downstream Promoter Element (DPE), Core Element Downstream (CED), TFIIB-Recognition Element (TRE), and Motif Ten Element (MTE) [20,27,28]. Together, these components act synergistically to increase transcription efficiency by providing recognition sites for transcription factors, and indicate the direction of transcription and also the DNA strand to be transcribed [20]. The transcription starts with the binding of the TFIID to the TATA-box region, the Inr sequence and/or other core promoter elements [27]. TFIID is a multiprotein complex comprising the TATA-box binding protein (TBP) and more than 10 different TBP associated factors (TAFs) [22]. After binding TBP to the TATA-box motif, the RNA pol II is recruited, and the transcription is triggered (Figure 1A).

Nevertheless, the transcription in eukaryotes is a much more complex process than previously thought and various strategies are used to increase the diversity of transcripts produced. Among mammals, previous analysis has shown that a large proportion of protein-coding genes (58%) use alternative promoters during transcription [25]. These alternative promoters may have different combinations of core promoter elements to increase the variability of transcripts [20,29,30].

There are many differences between the transcription process of eukaryotic and bacteria cells. The bacterial transcription is much simpler compared to the eukaryotic process since the transcription occurs using a single type of RNA pol and there are no transcription factors [31]. This enzyme is capable of synthesizing RNA from a DNA template, but it is unable to locate the promoter and transcription initiation site. Thus, a key factor to transcription is the free subunit named σ (sigma), which is responsible for recognizing the promoter region (Figure 1B) [32,33]. Although the majority of nucleotides within bacteria promoters vary in sequence, several short motifs are conserved. These include the hexamer (TATAAT), located 10 base pairs (bp) upstream of the TSS and is recognized by domain 2 of RNA pol σ subunit. Another motif is the the hexamer (TTGACA), located 35 base pairs (bp) upstream of the TSS and recognized by domain 4 of the RNA pol σ subunit [31,34,35]. In Archaea, there is a mix of eukarya and bacteria translational apparatus. Just as in eukaryotes, the archaea RNA pol is not able to recognize promoter sequences by itself and at least two transcription factors analogous to TBP and TFIIB are required [36,37,38]. The archeal TBP also recognizes specifically an AT-rich sequence, homologous to the TATA-box region of eukaryotes [39,40]. Although archaea transcription machinery is similar to that of eukaryotes, the characterization of transcription regulators of some archaeas showed that most of the transcriptional regulation in archaea is done by “bacterial-like” regulators, as two homologues of bacterial leucine-responsive regulatory protein (Lrp)—Lrs14 and Sa-Lrp and metal-dependent repressor 1 (MDR1) homologous to bacterial metal-dependent regulators (Figure 1C) [41,42,43].

Hypotheses regarding the evolutionary history of translational machinery among the living organisms have been raised during the last years, but the theme is still under debate [44]. Even considering the most recent proposals, the translational process of viruses remains out of the discussion, basically because these organisms are traditionally excluded from the canonical tree of life. However, this scenario has been changing since the discovery of giant viruses [16]. Therefore, it becomes interesting to examine if NCLDV members share similar transcription initiation strategies that could bring insights about how this correlates to giant viruses’ evolution.

### Gene Expression in NCLDVs

In contrast to cellular genomes, which are formed by dsDNA, viral genomes show a large diversity genome composition, structures, replication and transcription strategies with great implications in virus biology, as virus–host interactions [45]. The majority of the RNA viruses employ virus-coded specific enzymes (RNA-dependent RNA polymerases) to synthesize and modify their mRNA. DNA viruses showing small and intermediate size genomes such as the parvoviruses, papillomaviruses, and adenoviruses, depend on host-cell enzymes for transcription, including the RNA pol [45]. However, viruses with a large genome such as the giant viruses, mostly encode their transcriptional apparatus, which make them relatively independent from their host transcription machinery [15,46].

**Figure 2:**
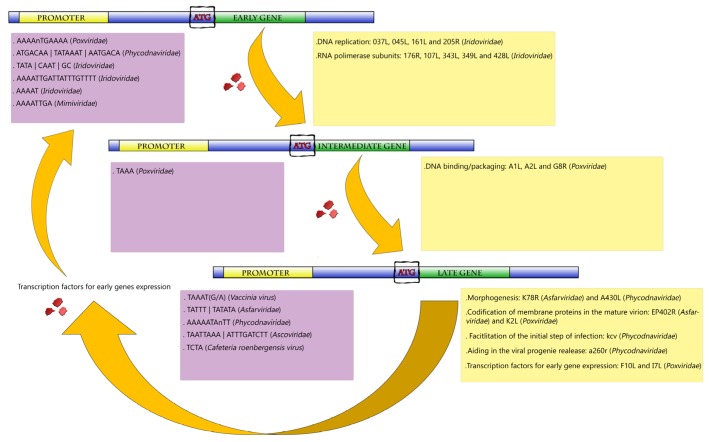
Representative scheme of the temporal gene expression in NCLDVs. During initial times of infection, the expression of genes related to the metabolism of nucleic acids is primarily activated (early and intermediate genes). After DNA replication, the activation of late genes is initiated. Those genes are involved in the production of viral structural proteins, in transcription factors used for early gene expression and also in proteins that facilitate the initial step of infection of the viral progeny in the next round of multiplication. Purple boxes represent the promoters described for giant viruses according to each gene category (early, intermediate and late genes). Yellow boxes exemplify the biological functions involved in each category, with some genes represented inside the parentheses.

The transcription of a typical large DNA virus occurs in a temporal pattern in the host cytoplasm (Figure 2). At the start of infection, a subset of immediate early viral proteins is required for DNA replication and host cell manipulation [47,48]. The early mRNAs also encode enzymes and factors needed for transcription of the intermediate genes. Concomitantly with the expression of intermediate genes, the expression of the early genes is often repressed. Finally, late genes are transcribed, directing the synthesis of structural proteins, non-structural proteins and enzymes present in the mature particle required for viral assembly [45,48]. The efficient transcription of late mRNA usually depends on intermediate gene products, as well as cellular transcription factors that may differ from those used by the early promoters. The products of the late genes include the immediate early transcription factors, which are packaged along with RNA pol and other enzymes within the virus progeny [47,48,49,50].

This ability to regulate temporally the transcription of genes is characterized as an evolutionary advantage. This strategy is possible due to the presence of promoter codes that dictate when, where, and at what level the classes of early, intermediate, and late genes are transcribed [45,48]. These promoter sequences are different between the three genes classes, but there is a pattern of conservation within the same group. This indicates that during the evolution the gene promoters were selected to ensure the temporal gene expression, and therefore ensure the gene expression in the host cell during its replication [45,47,48,50].

In the following sections, we look closer at how the gene transcription is carried out in each family of the proposed Megavirales order, focusing on the current knowledge about the promoter sequence of these viruses.

## 3. *Poxviridae* Family

Among NCLDVs, the *Poxviridae* family is one of the most studied. These viruses have enveloped ovoid particles of around 200 nm in diameter and 300 nm in length and present a linear dsDNA genome of approximately 200 kbp coding nearly 200 open reading frames (ORFs). Poxviruses can infect a wide range of hosts, such as insects, birds, and mammals [48,51]. Extensive study of the poxvirus genome and replication cycle allowed a detailed identification of its promoters, as well as important transcription factors. Poxviruses possess their own DNA-dependent RNA polymerase (RNA pol) that is very similar to the eukaryotic protein, regarding size and subunit complexity. In the case of *Vaccinia virus* (VACV), a poxvirus prototype, the enzyme subunits are encoded by eight viral VACV genes which, in most cases, are homologous to cellular RNApol [52,53]. Gene transcription in poxviruses follows a typical temporal profile regulated by well-conserved promoters of early, intermediate and late genes (Figure 2) [47,48].

**Figure 3:**
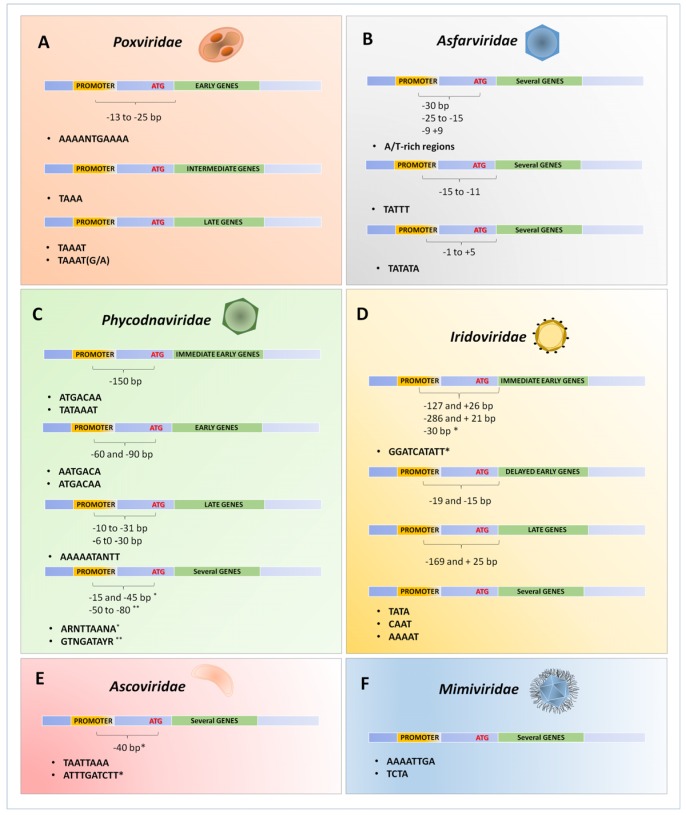
Schematic representation of the promoter’s sequences described for different NCLDVs. Compilation of the described promoters for some viral families belonging to the proposed order Megavirales: *Poxviridae* (**A**); *Asfarviridae* (**B**); *Phycodnaviridae* (**C**); *Iridoviridae* (**D**); *Ascoviridae* (**E**) and *Mimiviridae* (**F**). Each promoter was related to the expression of immediate early, early, delayed early, intermediate and late genes, or related to the expression of genes independent of temporal expression (several genes). The distances between the transcription start site or translate start site (ATG) until the promoters are also indicated by brackets.

The transcription of early genes is characterized by an A/T-rich motif upstream of transcriptional start site with a critical core region located from −13 to −25 to that region. Figure 3 illustrates the promoter motifs described in megavirales members. The representative consensus sequence of the early promoter region is ‘AAAANTGAAAA’. Mutagenesis in this promoter region of VACV causes a drastic negative effect on VACV gene transcription [54]. The intermediate genes are transcribed after DNA replication, before the transcription of the late genes. The intermediate core promoter is similar to the early promoter due to the A/T-rich content, but its specific sequence is given by the tetranucleotide ‘TAAA’. Furthermore, the intermediate promoter sequence has a bipartite structure presenting a core and an initiator region with similar sequences (TAAA) [55,56,57]. Three (*A1L*, *A2L*, and *G8R*) of the 53 genes that compose the set of intermediate genes encode transcription factors that are directly related to the late stage of the replication cycle, important to DNA binding/packaging processes and to core-associated proteins [58].

The transcription of late genes persists until the end of the replication cycle. Around 38 late genes have already been identified, with their main functions related to the codification of membrane proteins in the virion, morphogenesis steps, and also to the production of immediate early transcription factors [57,59]. Most of them are clustered in the central region of the poxviruses genome and also have A/T-rich sequence promoters. These regions consist of a core sequence of about 20 bp with some ‘T’ residues, separated by a region of about 5–7 bp of a conserved ‘TAAAT’ motif, which regulates the transcription initiation. Usually, G or A follows the late promoter sequence, performing a ‘TAAAT (G/A)’ transcription initiation sequence. This sequence is conserved among VACV late promoters, overlapping the site of transcription initiation that is absent in 5’ untranslated regions (5’-UTR) [48,54]. Mutations within this conserved element were demonstrated to cause complete inactivation of the promoter, and almost 25% of the ‘AAA’ sequences are used as transcription initiation sites in VACV. Along with other factors, the viral RNA pol directs the synthesis of late mRNAs, finishing the transcription process [54,60,61,62].

The presence of complete transcriptional machinery in poxviruses allows a lower dependency of these viruses on their hosts. It permits that the mRNA transcription totally occurs in the host’s cytoplasm, right after the virus entry. Addionally, the presence of well conserved promoter regulatory sequences in different poxviruses suggests a conserved evolutionary pattern among them. It is likely that such a complete transcriptional set was already present in their ancestor and was maintained over time. Alternatively, the presence of a robust transcriptional apparatus in all members of the *Poxivirdae* family might be a result of evolutive convergence. Although less parcimonious, the different poxviruses might have had different evolutionary histories regarding the transcription process, including both protein-related elements and promoter sequence regions, but in the course of evolution, they became more similar to each other. It is not yet possible to determine which hypothesis is the correct, or even if other possibilities correspond to the real history of these complex viruses, and this discussion shall continue for a while.

## 4. *Asfarviridae*

*African swine fever virus* (ASFV), a large (~200 nm), icosahedral, and enveloped virus is currently the single member of the *Asfarviridae* family, infecting members of the *Suidae* family (pigs, hogs and boars) [63]. The genome is composed of a linear dsDNA molecule of approximately 170 kbp with terminal inverted repeats. It encodes approximately 150 ORFs separated by short intergenic regions [64,65]. ASFV encodes its own RNA pol and all ASFV genes are transcribed by its enzyme [66,67].

Similar to poxviruses, the ASFV gene transcription follows a temporal profile, where immediate early and early genes are expressed before the DNA replication that is followed by the expression of intermediate, late and immediate early genes. Transcription initiation and termination occurs at very precise positions in the genome, encoding a several genes involved in the transcription and modification of viral mRNAs. The transcriptional machinery of ASFV provides an accurate temporal control of gene expression regulated by cis-DNA elements, enhancers, and promoters together with a structural complexity of transcription factors [68]. Analysis of the base composition of the intergenic regions shows that they are rich in A/T sequences, similar to that observed in poxviruses [69,70,71]. A/T-rich regions located at approximately −30 bp upstream of the ATG translation start site are essential for the expression of the K9L gene, which encodes a protein with similarity to mammalian transcription elongation factor IIS [72]. Furthermore, upstream sequences presented in two intermediate genes exhibit highly conserved sequences at positions −25 to −15, and −9 to +9 to the translational start codon [70]. Experiments involving genetic deletions, linker scan substitutions and point mutations in the promoter sequence of the *p72* gene (major capsid protein) revealed that the replacement of the A/T-rich region by G/C residues strongly reduced the transcription rate, demonstrating the importance of this sequence for efficient late viral transcription [71].

Two other major essential regions for promoter activity are described: one region is located at position −15 to −11 upstream of the transcription start site (TATTT); and the second region at positions −1 to +5 (TATATA) [71]. Mutants presenting the ‘TATATA’ motif replaced by a G/C-rich sequence had the promoter activity completely abolished, suggesting that ASFV transcription is dependent on such sequence at (or near) the region of transcriptional initiation, similar to what is found in other large viruses [71]. The replacement of the equivalent ‘TATATA’ sequence on the late genes *K78R*, *EP402R* and *A137R* by the ‘GCGC’ motif was also demonstrated to be deleterious, suggesting that the A/T-rich sequence could be a motif for late promoter function as well [68,71]. Interestingly, the bipartite structure seen in the late promoter of ASFV is similar to the late and intermediate promoters in poxviruses that contain a core and an initiator region [54,55,62,71]. The similarities found in the transcriptional strategies reinforce the genetic data, indicating a close relationship between poxviruses and asfavirus, pointing to a common ancestor for both viral families.

## 5. *Phycodnaviridae*

The phycodnaviruses are large and icosahedral viruses (~100–220 nm), with dsDNA genomes ranging from 180 to 560 kbp [73]. Since they infect a diverse group of eukaryotic algae, they are one of the most important groups of organisms regulating the oxygen cycle in the Earth [74,75]. The family *Phycodnaviridae* consists of six genera, named according to the hosts that they infect: *Chlorovirus*, *Coccolithovirus*, *Prasinovirus*, *Prymnesiovirus*, *Phaeovirus*, and *Raphidovirus* [76]. As demonstrated by other giant viruses, the phycodnaviruses exhibit a temporal transcription profile. Early genes are transcribed within 5 to 60 min post-infection (p.i), and transcripts of late genes begin to appear around 60–90 min p.i. However, some early genes can also be detected in later stages of infection [77,78].

The presence of A/T-rich promoters was also observed in phycodnaviruses. Analysis of the *kcv* gene, encoding a potassium ion channel protein in chlorella viruses, revealed a highly conserved 10-nt sequence (AAAAATANTT) in the promoter region of this gene, present in 16 out of 17 chlorellaviruses [77]. This sequence is located at 10–31 nucleotides upstream of the ATG translation start codon in all of the analyzed viruses, and it was associated with late gene transcription, since, apparently, *kcv* transcripts are produced during the late steps of infection. Furthermore, the region that precedes seven genes expressed at later times during the *Paramecium bursaria chlorella virus 1* (PBCV-1) replication cycle (*a85r*, *a237r*, *a248r*, *a260r*, *a292l*, *a430l*, and *a530r*) contain the same sequence or at least a subset of this sequence located at 6–30 nucleotides upstream of the ATG start codon [77]. The study of immediate early genes expressed in chlorovirus infections also revealed A/T-rich sequences as putative promoter regions. Two sequences determined by ‘ATGACAA’ and ‘TATAAAT’ (such as the eukaryotic “TATA-box”) were located in a 150 bp region from the translation start codon in the upstream regions of almost all immediate early genes (20 of 23 studied) [78]. These elements, especially ‘ATGACAA’, were absent in all genes so far examined, expressed after 40 min p.i, including *A122R* (Vp260) [79], *A181-182R* (chitinase), *A292L* (chitosanase) [80], *A430L* (major capsid protein) [81], *vAL-1* [82].

Bioinformatics analysis revealed highly conserved nucleotide sequences in putative promoter regions involving three different chlorella viruses: PBCV-1, virus MT325 [83], and *Paramecium bursaria chlorella virus* NY-2A [84]. Three putative AT-rich sequence promoters, comprising seven to nine nucleotides (ARNTTAANA, AATGACA and GTNGATAYR), located at 150-nt upstream of the translation start codon of many ORFs were observed [85]. The ‘ARNTTAANA’ sequence is found between nucleotides −15 and −45 relative to the ATG translation start codon. This sequence occurs in the promoter region of 25% of PBCV-1 genes, 22% of NY-2A genes and 12% of MT325 genes. Regarding the entire genome, this sequence is present within the 200-nt promoter region during 44% of the time in PBCV-1, 49% of the time in NY-2A, and 37% of the time in MT325. The hotspot for the presence of the ‘AATGACA’ sequence is located between nucleotides −60 and −90 from the translational start codon. This sequence occurs in the promoter region of 16% of the PBCV-1 genes, 18% of NY-2A genes and 8% of MT325 genes. Regarding the entire genome, this sequence is present within the 200-nt promoter region in 54% of the PBCV-1 genes, 53% of the NY-2A genes, and 25% of the MT325 genes [85]. The ‘AATGACA’ sequence in PBCV-1 is associated with early genes during the replication cycle [85]. This sequence is very similar to a motif previously identified in some chlorella viruses (ATGACAA), which is also correlated with early transcripts [78]. Finally, the ‘GTNGATAYR’ sequence is mainly located at nucleotide positions −50 to −80 from the ATG initiation codon, occurring in the promoter region of 13% of PBCV-1 genes, 14% NY-2A genes, and in 11% of MT325 genes. Regarding the entire genome, this sequence is found specifically within the 200-nt promoter region in 28% of the PBCV-1 genes, 22% of the NY-2A genes, and 21% of the MT325 genes [85].

Unlike other members of the NCLDVs, phycodnaviruses do not encode their own RNA pol and need to appropriate the host’s RNA pol to properly make their transcripts [86]. However, uniquely for the *Phycodnaviridae* family, *Emiliania huxleyi virus 86* (EhV-86), a coccolithovirus that infects the marine calcifying microalga *Emiliania huxleyi*, contains a total of six RNA pol subunits, which suggests that this virus partially encodes its own transcription machinery [87]. Although these viruses present some important elements for the mRNA synthesis, it is not possible to state that they have their own transcriptional complete apparatus, at least for the majority of them. Therefore, concerning the transcriptional process, the phycodnaviruses seem to present a different evolutionary history.

## 6. *Iridoviridae*

The *Iridoviridae* family is composed by five genera: *Ranavirus*, *Megalocytivirus* and *Lymphocystivirus* that infect vertebrates; *Iridovirus* and *Chloriridovirus* that infect invertebrates [88]. Iridoviruses have a linear dsDNA genome varying from 105 to 212 kbp, coding between 92 and 211 putative proteins. They present a non-enveloped icosahedral particle of 300 nm in size [89,90,91,92]. These large viruses also display a pattern of temporal gene expression regulation, wherein the genes are divided into three classes: immediate-early (IE or α), delayed-early (DE or β), and late (L or γ) genes [93,94,95]. Iridoviruses are typical nucleo-cytoplasmic viruses. They begin the replication cycle in the nucleus, followed by the second phase of genome replication in the cytoplasm [90].

Gene transcription and promoter sequences studies have been performed for only a few genes in members of the *Iridoviridae* family. The study of promoter sequences in iridovirus is focused mainly in the *Ranavirus* genus (using type species *Frog virus 3* (FV3)) and *Iridovirus* genus (using type species *Invertebrate iridescent virus 6* (IIV-6)), the type species of the *Iridovirus* genus. Notwithstanding, both the gene expression and promoter sequences studies have been performed for only a few genes in the *Iridoviridae* family. The most complex studies were performed with immediate-early *ICR-169* and *ICR-489* genes of FV3 [96,97]. Those studies revealed the importance of a 78 bp sequence before the transcription start site of an IE gene of the FV3 promoter. It was shown that an FV3 protein acts in trans to induce the transcription of the major FV3 IE gene, *ICR-169*, and is dependent on the 78 bp sequence located at the 5′ position from the start site of the transcription of this gene [98]. Two years later, the same group demonstrated that a 23 bp sequence was possibly a critical *cis*-regulatory element for the occurrence of FV3 *trans*-activation, since a significant reduction of transcription occurred after its deletion, located at the 5′ region, showing the sequence ‘ATATCTCACAGGGGAATTGAAAC’ [96]. Despite the importance of the approximately 23-nt sequence upstream of the transcription start site in the IE *ICR-169* gene of FV3, this sequence had no similarity with the promoter region of the intermediate gene *ICR489*. This lack of similarity indicated that the contemporary regulation of these two promoters is not controlled by sequences upstream of the start point of transcription [97]. It is worthy to note that in the *ICR489* gene, in an upstream region, ‘TATA’, ‘CAAT’, and ‘GC’ motifs were identified, which are similar to those of typical eukaryotic promoters [97].

Another study analyzed three genes—two early (*ICP-18* and *ICP-46*) and a late one [major capsid protein (MCP)] of *Bohle iridovirus* (another *Ranavirus* member)—looking for conserved regions to be considered as regulatory elements [99]. The authors demonstrated that all gene promoters included sequences located 127 to 281 bases upstream of the transcription initiation site (127 pb or ICB-18, 281 pb for ICP46, and 169 pb for MCP), but also sequences located from 21 to 26 bases downstream of this site (26 bases for *ICP-18*, 21 bases for ICP 46 and 25 bases for MCP) [99].

Moreover, a detailed study conducted in the following years identified an essential ‘AAAAT’ motif in a DE gene of IIV-6 (*Iridovirus*) [100]. The authors described a sequence of 19 bp (AAAATTGATTATTTGTTTT), located between −19 and −2 relative to the mRNA transcription start site, which is the putative region responsible for promoter activity of the DNApol gene. Deletions and point mutations in the DNApol promoter of IIV-6 showed that each of the 5-nt of ‘AAAAT’ motif located between −19 and −15 were equally essential for promoter activity. Mutations at the downstream side had a lower effect, but the role of individual nucleotides positioned at −14 to −5 was not analyzed in this study [100].

It is noteworthy that the same critical ‘AAAAT’ motif was found in the 100-nt upstream of the putative translational start codons of several other putative DE IIV-6 genes [91]. In *Invertebrate iridescent virus 3* (IIV-3), many homologues of these genes also presented the ‘AAAAT’ motif in proximity to their start codon. A great similarity was also found between the region upstream of the DNApol ORF and the corresponding region in 12 iridovirus genomes [101]. Eight of these genomes showed a similar ‘AAAAT’ motif in the DNApol upstream region and three sequenced ranavirus genomes also shared the related ‘TAAAT’ motif in their DNA pol promoter region, which may indicate a conserved regulation of DE promoter activity in iridoviruses [101].

A study that targeted a IE gene (012L) of IIV-6 showed that the transcription start site is located 30-nt upstream of the ATG translational start codon. Analyzing mutants (produced by deletion), it was established that the intergenic region located between −21 and −10 (GGATCATATT) upstream of the transcription start site comprised the promoter sequence promoter 012L gene. This type of sequence was not observed in upstream regions of other IE genes of IIV-6, such as 468R, 006L and 010R. The ‘TATA’ and ‘CAAT’ sequences were also identified in the intergenic region of this gene, as well as sequences similar to the ‘AAAAT’ motif described to the DNA pol gene, but this sequence had no promoter activity for the 012L, differently than demonstrated for the DNA pol gene. The 037L and 012L genes of IIV-6, both early genes, do not share conserved key promoter motifs. However, DNA pol is considered a DE gene and 012L an IE gene [100,102].

Despite the presence of homologs of RNA pol subunits in the iridoviruses genome, host RNA pol II is required for the synthesis of *Ranavirus* IE transcripts, and it is likely that the same is true from *Iridovirus* IE genes, contrasting to pox- and asfaviruses [103,104,105,106]. It has been proposed that the RNA pol subunits found in members of the *Iridoviridae* family are probably involved in the cytoplasmic phase of transcription in later stages of infection [91,107]. Such a paradox may reflect the long co-evolution period that these viruses had been through. It is possible that the ancestor of iridoviruses presented a complete transcription apparatus, but some elements were lost due to the adaptation to a more parasitic lifestyle. Other possibilities are the occurrence of events of horizontal gene transfer (HGT) between the viruses and their hosts. However, the lack of information about such events involving members of the *Iridoviridae* family prevents further insights into this alternative for the evolution of the transcription apparatus of these viruses.

## 7. *Ascoviridae*

The *Ascoviridae* family has two genera that include *Ascovirus*, with three species including *Spodoptera frugiperda ascovirus 1a* (SfAV-1a), the prototype of the genus, *Trichoplusia ni ascovirus 2a* (TnAV-2a), and *Heliothis virescens ascovirus 3a* (HvAV-3a), and the *Toursvirus* genus, with only one representative, *Diadromus pulchellus ascovirus 4a* (DpAV-4a) [108,109]. Ascoviruses are enveloped viruses, 300–400 nm long by 100–150 nm in diameter, with a circular dsDNA genome with sizes ranging from 116 to 185 kb, infecting arthropods, mainly lepidopterans [110,111,112].

The studies regarding the ascoviruses are still in their infancy. Information about the replication and more specifically, the transcription process, are extremely scarce. The current knowledge about transcription in ascoviruses come from the analyses of the *Ascovirus* genus [110,113]. A study performed using a possible variant of HvAV-3, the *Spodoptera exigua ascovirus 5a* (SeAV-5a) showed that the 5’-UTR region of the SeAV-5a MCP gene is composed of 25-nt [114]. The upstream region of this gene does not present a typical eukaryotic class II promoter motif sequence ‘TATAAAT’ (TATA box). However, the putative 5’ transcription control region of the SeAV-5a MCP gene shares similarities with other ascoviruses and iridoviruses, containing a conserved TATA-box like motif (TAATTAAA) and an ‘ATTTGATCTT’ motif within 40-nt upstream of the translation initiation codon ATG [114]. The ‘TAATTAAA’ and ‘ATTTGATCTT’ motifs are located downstream and upstream of the transcription initiation site, respectively. Furthermore, the ORF p27 presents a similar 5’ downstream transcription promoter region, suggesting that such a region might be a truly regulatory sequence within ascoviruses [114].

Sequences from the promoter regions of the MCP genes from ascoviruses and IIV-6 (late genes), showed that ascoviruses and iridoviruses are closely related in this aspect, suggesting that the transcription regulation could be maintained during the viral evolution process in closely related viruses [115,116]. Furthermore, phylogenetic studies showed that ascoviruses probably evolved from the iridoviruses [116,117,118]. It is possible that the same pattern of temporary gene expression exhibited in iridoviruses (and the other members of proposed Megavirales order) was conserved in the ascoviruses lineage, and that such a mechanism might have been present in their common ancestor.

## 8. *Mimiviridae* and Other Amoebal Giant Viruses

The discovery of mimiviruses in 2003 and the establishment of the *Mimiviridae* family astonished the scientific community, making the term ‘giant virus’ more appropriated than ever. These viruses have particles visible in light microscopy, with sizes of ~700 nm in diameter. Viral particles have characteristics never described before in the virosphere, such as long proteic fibrils (~125 nm in length) immersed in a peptidoglycan matrix, and a star-shaped face, named stargate, responsible for the releasing of the genome inside the cytoplasm of their host (*Acanthamoeba* genus) [4,119,120,121]. The genome is a linear dsDNA molecule of about 1.2 Mbp, coding more than 1000 proteins, including a large set of transcriptional elements [15,122].

Similar to other NCLDVs members, mimiviruses genes can be divided into early, intermediate and late categories according to three major temporal classes of transcription determined by mRNA deep sequencing [49]. The analysis of the intergenic regions of *Acanthamoeba polyphaga mimivirus*, the prototype species of *Mimivirus* genus, showed a conserved ‘AAAATTGA’ motif in nearly 50% of genes [50]. The intergenic regions of the genome of mimiviruses have an average size of 157-nt. In silico analyses showed that the conserved ‘AAAATTGA’ motifs are present within the 150-nt upstream regions of the translation start codon in 45% of all predicted mimivirus genes [50]. This motif is mainly associated to early (or the late-early) genes during the viral infectious cycle, and it is absent from the upstream regions of mimivirus late genes, such as DNA replication and particle morphogenesis and assembly. It is noteworthy that similar sequences were described regulating the early genes in other giant viruses, such as iridoviruses and phycodnaviruses, as described in the topics above. Besides the early promoter sequence, another A/T-rich motif (two 10-nt informative segments separated by a highly degenerated 4-nt sequence) was identified as a putative late promoter within mimiviruses, which is present in 24.2% of the considered late class genes. To the best of our knowledge, an intermediate promoter sequence has not already been described in mimiviruses [49,50].

In a distant relative, the *Cafeteria roenbergensis virus* [CroV (*Cafeteria* genus)], *Mimiviridae* family; the same early promoter motif was identified in the upstream region of 35% of genes [123]. However, considering the late promoter motif, this virus exhibits a different putative regulatory sequence compared to other mimiviruses, wherein the ‘TCTA’ tetramer flanked by A/T-rich regions on either side was found in the 5’ upstream of 124 late genes [123]. Moreover, CroV present eight RNA pol II subunits, six transcription factors, several helicases, among others, indicating the presence of nearly complete transcriptional machinery. This feature seems to be a mark to all members of the *Mimiviridae* family, which suggests that such a robust transcriptional apparatus was already present in the last common ancestor.

After the discovery of mimiviruses, other giant viruses infecting amoebae were described, such as marseilleviruses, which is currently classified in the family *Marseilleviridae* [124]. Other viruses have also been isolated but still not properly classified, namely faustoviruses [125], pandoraviruses [8,126], phitoviruses [127,128] and mollivirus [129]. Although these viruses are not yet officially recognized by the ICTV, they are genuine members of the NCLDVs [6,7,9]. In all of these giant viruses, a set of transcriptional elements has already been identified, including many RNA pol subunits, indicating a nearly autonomous process in these viruses. However, analysis of promoters and studies aiming to understand how gene expression is regulated in those newly discovered viruses remain to be performed.

## 9. MEGA-Box: A Putative Promoter Region in the Common Ancestor of Megavirales

The proposed Megavirales order comprises viral families that exhibit some unique features that allow their clustering into a monophyletic group [5]. In addition to some core genes that are shared among these viruses, they present other similarities, such as a temporal transcription profile. As described above, all viruses present elements to the transcriptional apparatus, most of them reaching up to the independence from their host in this step of the viral life cycle. Also, the presence of an A/T-rich promoter sequence has been described in many representatives of each family, even in those in which the genome presents a high G/C content. More interesting is the fact that some promoter sequences found in one family are very similar to others found in their relatives (Figure 3). This fact suggests that a possible common ancestor of the Megavirales order likely had an A/T-rich promoter sequence. More interesting is the fact that some promoter sequences found in one family are very similar to others found in their giant relatives. This fact suggests that such a common ancestor of Megavirales likely had an A/T-rich promoter sequence.

The origin of the members of the Megavirales order is still under debate, but the evolutionary history of some of its members is already being told, at least concerning genome evolution. The first members to be analyzed were the poxviruses. It has been demonstrated by phylogenetic analysis based on the presence/absence of genes that genomes from this family have been subject to frequent events of gene duplication, deletion, and HGT from their hosts. Many of these genes can interfere with host immune signaling, such as homologues of cytokines receptors which could confer some advantages in the interaction with the hosts [130,131,132]. By analyzing the poxviruses’ closest relative, ASFV, it seems that it has been through the same pattern of evolution, at least considering the multigene and p22 gene families [133,134].

The “accordion-like” pattern of evolution was also identified in different members of the *Iridoviridae* family. It is particularly interesting the fact that iridoviruses infecting the same host-range exhibited a similar pattern of gene gain and loss, but this was slightly different when the viruses infected different hosts (fish vs. insect-infecting viruses), suggesting that such a pattern was driven by host–virus co-evolution [135]. Finally, the same evolutionary model for members of the families *Phycodnaviridae* and *Mimiviridae* has recently been described. The genomic comparisons of closely related viruses belonging to the *Mimiviridae* and *Phycodnaviridae* families show that genomes accumulating genomic mutations occur on successive cycles of genome expansion and reduction. In addition, there is no general tendency of genome expansion or contraction. Each family exhibits a specific pattern for gene acquisition, which might be a reflex of interaction with distinct hosts [10]. Since these viruses seem to exhibit a similar pattern of genome evolution, it is possible that a similar scenario has also happened with their promoter sequences. In the same way, it is reasonable to consider that NCLDVs’ common ancestor evolved by the same “accordion-like” pattern, and thus it presented a promoter region that underwent an analogous mechanism.

Considering a common origin for the NCLDVs, a possible scenario is that the Megavirales’ common ancestor presented a ‘TATATAAAATTGA’ promoter motif, which we named here as the “MEGA-box” (an allusion to the conserved TATA-box promoter found in cellular organisms). Over time, with the Megavirales’ order radiation, the MEGA-box has been gradually evolved by nucleotides’ gain and loss, analogously to that reported for the entire genome, which evolved through gene gain and loss. The MEGA-box was slightly modified in the poxviruses lineage, at least concerning the early promoter motif. Considering the intermediate and the late promoter motifs of poxviruses, if they truly came from the MEGA-box, this could have happened through a series of nucleotide loss. However, it is also possible that the emergence of other promoters, rather than the early one, have emerged after the establishment of the poxvirus’ lineage, thus not originating from the ancestral promoter sequence. The same might be true for mimiviruses, phycodnaviruses and iridoviruses. Considering asfavirus and ascoviruses, their promoter sequences might have originated from the MEGA-box through successive gain and loss of nucleotides. However, another scenario is also possible, wherein their promoter motifs emerged from the poxviruses and iridoviruses lineages respectively (closest evolutionary groups). This scenario is in agreement with the proposition that the Megavirales’ ancestor was already a giant virus with a large genome [10]. In this aspect, the giant ancestor also had a large promoter sequence that evolved through constant nucleotide gain and loss, a pattern analogous to the accordion-like model of genome evolution. However, other scenarios are also possible, although less probable, considering the evolutionary data currently available for these viruses. One is that the ancestor had a very short promoter sequence, like a poxvirus intermediate promoter (TAAA), that underwent massive nucleotide gain over time, leading to very large promoter sequences in the majority of the giant viruses. Another one is just the opposite; wherein the ancestor had a very large promoter region that had been losing nucleotides during evolution. A third pathway, equally unlikely, would be the acquisition of promoter sequences by horizontal/lateral transfer. Similar to different genes, the MEGA-box promoter evolutionary pattern during the radiation of NCLDVs members could be related to the co-evolution with different hosts over time.

Whether the NCLDVs came from a simple entity [14,136], or from an already complex organism [10,16,137], is still under debate. Despite this, increasing evidence that they originated from a common ancestor is emerging, and it suggests that such an ancestor evolved through an “accordion-like” pattern. By analyzing the promoter regions currently known for different giant viruses, we provide another piece of evidence to support this statement. Further, we propose how a conserved A/T-rich promoter sequence was present in the possible common ancestor, which might have evolved by continuous gain and loss of nucleotides, in addition to some point mutations in the MEGA-box original sequence. Other scenarios could also be discussed for the evolution of the promoter sequences of the NCLDVs, including selective sweep or convergence. However, these alternatives run off the diffused hypothesis of a common origin for the putative Megavirales order.

## 10. What Comes Next?

Most of the giant viruses have a powerful genetic arsenal, encoding several proteins necessary for the transcription system which provides a relative independence of their hosts for this process. In addition, the transcription of this high gene content is temporally regulated by promoter regions that exhibit some similarities, indicating a common origin of these regulatory elements. Although many studies have already been done in relation to almost all viral families of the Megavirales order, most of them remain without biological confirmation; i.e., the promoter motifs in many giant viruses were predicted, but not experimentally validated. Therefore, the performance of biological studies to confirm the existence and the effect of all promoter motifs described so far in giant viruses is imperative. This analysis will truly establish the common temporal regulation pattern predicted in these viruses, and will also corroborate (or even refute) the hypothesis of an A/T-rich promoter in the Megavirales common ancestor. Moreover, the deep analysis of the genome of the recently described giant viruses (Marseilleviruses, Pandoraviruses, Pithoviruses, Faustoviruses and Mollivirus), and also the discovery of new complex viruses, will strongly contribute to complete the puzzle of the origin and evolution of Megavirales.

On the other hand, the biotechnology field will also be boosted by the advance in the studies of promoters and gene expression in giant viruses. Among the NCLDVs, the poxviruses are by far the best characterized group regarding the genome expression, especially the VACV. These viruses have been used as expression vectors for the synthesis of proteins and as vaccine candidates to prevent infectious diseases and treat cancer, mainly due to their high gene expression levels [69,138]. This attribute is clearly shared with other giant viruses that were recently described, and the real comprehension of their gene regulation and expression will bring uncountable possibilities for biotechnology purposes. Finally, the impact of the giant viruses on the basic comprehension of the origin and evolution of life is undeniable, as well as for their ecological, medical and technological importance. The discovery of even more complex viruses associated with the advance of many techniques used for genomic studies will certainly answer those remaining questions around the NCLDVs, and will surely bring new exciting challenges for the whole scientific community.

## References

[1] Lwoff A. (1957). The concept of virus. J. Gen. Microbiol..

[2] Durzyńska J., Goździcka-Józefiak A. (2015). Viruses and cells intertwined since the dawn of evolution. Virol. J..

[3] Iyer L.M., Aravind L., Koonin E.V. (2001). Common origin of four diverse families of large eukaryotic DNA viruses. J. Virol..

[4] La Scola B., Audic S., Robert C., Jungang L., de Lamballerie X., Drancourt M., Birtles R., Claverie J.M., Raoult D. (2003). A giant virus in amoebae. Science.

[5] Colson P., de Lamballerie X., Fournous G., Raoult D. (2012). Reclassification of giant viruses composing a fourth domain of life in the new order Megavirales. Intervirology.

[6] Sharma V., Colson P., Chabrol O., Pontarotti P., Raoult D. (2015). *Pithovirus sibericum*, a new bona fide member of the “Fourth TRUC” club. Front. Microbiol..

[7] Sharma V., Colson P., Chabrol O., Scheid P., Pontarotti P., Raoult D. (2015). Welcome to pandoraviruses at the “Fourth TRUC” club. Front. Microbiol..

[8] Scheid P. (2016). A strange endocytobiont revealed as largest virus. Curr. Opin. Microbiol..

[9] Benamar S., Reteno D.G., Bandaly V., Labas N., Raoult D., la Scola B. (2016). Faustoviruses: Comparative genomics of new Megavirales family members. Front. Microbiol..

[10] Filée J. (2015). Genomic comparison of closely related Giant Viruses supports an accordion-like model of evolution. Front. Microbiol..

[11] Suzan-Monti M., la Scola B., Raoult D. (2006). Genomic and evolutionary aspects of Mimivirus. Virus Res..

[12] Claverie J.M. (2006). Viruses take center stage in cellular evolution. Genome Biol..

[13] Moreira D., López-García P. (2005). Comment on “The 1.2-megabase genome sequence of Mimivirus”. Science.

[14] Yutin N., Wolf Y.I., Koonin E.V. (2014). Origin of giant viruses from smaller DNA viruses not from a fourth domain of cellular life. Virology.

[15] Raoult D., Audic S., Robert C., Abergel C., Renesto P., Ogata H., la Scola B., Suzan M., Claverie J.M. (2004). The 1.2-megabase genome sequence of Mimivirus. Science.

[16] Boyer M., Madoui M.A., Gimenez G., La Scola B., Raoult D. (2010). Phylogenetic and phyletic studies of informational genes in genomes highlight existence of a 4 domain of life including giant viruses. PLoS ONE.

[17] Schones D.E., Cui K., Cuddapah S., Roh T.-Y., Barski A., Wang Z., Wei G., Zhao K. (2008). Dynamic regulation of nucleosome positioning in the human genome. Cell.

[18] Fuda N.J., Ardehali M.B., Lis J.T. (2009). Defining mechanisms that regulate RNA polymerase II transcription in vivo. Nature.

[19] Lacadie S.A., Ibrahim M.M., Gokhale S.A., Ohler U. (2016). Divergent transcription and epigenetic directionality of human promoters. FEBS J..

[20] Haberle V., Lenhard B. (2016). Promoter architectures and developmental gene regulation. Semin. Cell Dev. Biol..

[21] Weiss S., Gladstone L. (1959). A mammalian system for the incorporation of cytidine triphosphate into ribonucleic acid. J. Am. Chem. Soc..

[22] Thomas M.C., Chiang C.M. (2006). The general transcription machinery and generalcofactors. Crit. Rev. Biochem. Mol. Biol..

[23] Sabin L.R., Delás M.J., Hannon G.J. (2013). Dogma derailed: The many influences of RNA on the genome. Mol. Cell.

[24] Vannini A., Ringel R., Kusser A.G., Berninghausen O., Kassavetis G.A., Cramer P. (2010). Molecular basis of RNA polymerase III transcription repression by Maf1. Cell.

[25] Carninci P., Sandelin A., Lenhard B., Katayama S., Shimokawa K., Ponjavic J., Semple C.A., Taylor M.S., Engström P.G., Frith M.C. (2006). Genome-wide analysis of mammalian promoter architecture andevolution. Nat. Genet..

[26] Cooper S.J., Trinklein N.D., Anton E.D., Nguyen L., Myers R.M. (2006). Comprehensive analysis of transcriptional promoter structure and functionin 1% of the human genome. Genome Res..

[27] Smale S.T., Kadonaga J.T. (2003). The RNA polymerase II core promoter. Annu. Rev. Biochem..

[28] Maston G.A., Evans S.K., Green M.R. (2006). Transcriptional regulatory elements in the human genome. Annu. Rev. Genom. Hum. Genet..

[29] Hansen S.K., Tjian R. (1995). TAFs and TFIIA mediate differential utilization of thetandem Adh promoters. Cell.

[30] Ren B., Maniatis T. (1998). Regulation of Drosophila Adh promoter switching by aninitiator-targeted repression mechanism. EMBO J..

[31] Browning D.F., Busby S.J. (2016). Local and global regulation of transcription initiation in bacteria. Nat. Rev. Microbiol..

[32] Burgess R.R., Travers A.A., Dunn J.J., Bautz E.K. (1969). Factor stimulating transcription by RNA polymerase. Nature.

[33] Lee D.J., Minchin S.D., Busby S.J. (2012). Activating transcription in bacteria. Annu. Rev. Microbiol..

[34] Murakami K.S., Masuda S., Campbell E.A., Muzzin O., Darst S.A. (2002). Structural basis of transcription initiation: An RNA polymerase holoenzyme–DNA complex. Science.

[35] Browning D.F., Busby S.J. (2004). The regulation of bacterial transcription initiation. Nat. Rev. Microbiol..

[36] Hausner W., Wettach J., Hethke C., Thomm M. (1996). Two transcription factors related with the eucaryal transcription factors TATA binding protein and transcription factor IIB direct promoter recognition by an archaeal RNA polymerase. J. Biol. Chem..

[37] Bell S.D., Jaxel C., Nadal M., Kosa P.F., Jackson S.P. (1998). Temperature template topology, and factor requirements of archaeal transcription. Proc. Natl. Acad. Sci. USA.

[38] Darcy T.J., Hausner W., Awery D.E., Edwards A.M., Thomm M., Reeve J.N. (1999). Methanobacterium thermoautotrophicum RNA polymerase and transcription in vitro. J. Bacteriol..

[39] Reiter W.D., Hudepohl U., Zillig W. (1990). Mutational analysis of an archae bacterial promoter—Essential role of a TATA Box for transcription efficiency and start-site selection in vitro. Proc. Natl. Acad. Sci. USA.

[40] Palmer J.R., Daniels C.J. (1995). In vivo definition of an archaeal promoter. J. Bact..

[41] Kyrpides N.C., Ouzounis C.A. (1999). Transcription in Archaea. Proc. Natl. Acad. Sci. USA.

[42] Aravind L., Koonin E.V. (1999). DNA-binding proteins and evolution of transcription regulation in the *Archaea*. Nucleic Acids Res..

[43] Bell S.D., Jackson S.P. (2001). Mechanism and regulation of transcription in *Archaea*. Curr. Opin. Microbiol..

[44] Werner F., Grohmann D. (2011). Evolution of multisubunit RNA polymerases in the three domains of life. Nat. Rev. Microbiol..

[45] Whelan S., Knipe D.M., Howley P.M. (2014). Viral Replication Strategies. Fields Virology.

[46] Abergel C., Rudinger-Thirion J., Giege R., Claverie J.M. (2007). Virus-encoded aminoacyl-tRNA synthetases: Structural and functional characterization of mimivirus TyrRS and MetRS. J. Virol..

[47] Broyles S.S., Knutson B.A. (2010). Poxvirus transcription. Future Virol..

[48] Moss B., Knipe D.M., Howley P.M. (2014). Poxviridae. Fields Virology.

[49] Legendre M., Audic S., Poirot O., Hingamp P., Seltzer V., Byrne D., Lartigue A., Lescot M., Bernadac A., Poulain J. (2010). mRNA deep sequencing reveals 75 new genes and a complex transcriptional landscape in Mimivirus. Genome Res..

[50] Suhre K., Audic S., Claverie J.M. (2005). Mimivirus gene promoters exhibit an unprecedented conservation among all eukaryotes. Proc. Natl. Acad. Sci. USA.

[51] Damon I.K., Knipe D.M., Howley P.M. (2014). Poxviruses. Fields Virology.

[52] Baroudy B.M., Moss B. (1980). Purification and characterization of a DNA dependent RNA polymerase from vaccinia virions. J. Biol. Chem..

[53] Knutson B.A., Broyles S.S. (2008). Expansion of poxvirus RNA polymerase subunits sharing homology with corresponding subunits of RNA polymerase II. Virus Genes.

[54] Davison A.J., Moss B. (1989). The structure of vaccinia virus early promoters. J. Mol. Biol..

[55] Baldick C.J., Keck J.G., Moss B. (1992). Mutational analysis of the core, spacer and initiator regions of vaccinia virus intermediate class promoters. J. Virol..

[56] Knutson B.A., Liu X., Oh J. (2006). Vaccinia virus intermediate and late promoter elements are targeted by the TATA-binding protein. J. Virol..

[57] Yang Z., Bruno D.P., Martens C.A., Porcella S.F., Moss B. (2011). Genome-wide analysis of the 5’and 3’ends of vaccinia virus early mRNAs delineates regulatory sequences of annotated and anomalous transcripts. J. Virol..

[58] Keck J.G., Baldick C.J., Moss B. (1990). Role of DNA replication in vaccinia virus gene expression: A naked template is required for transcription of three late transactivator genes. Cell.

[59] Yang Z., Reynolds S.E., Martens C.A., Bruno D.P., Porcella S.F., Moss B. (2011). Expression profiling of the intermediate and late stages of poxvirus replication. J. Virol..

[60] Yang Z., Martens C.A., Bruno D.P., Porcella S.F., Moss B. (2012). Pervasive initiation and 3’-end formation of poxvirus postreplicative RNAs. J. Biol. Chem..

[61] Broyles S.S., Liu X., Zhu M., Kremer M. (1999). Transcription factor YY1 is a vaccinia virus late promoter activator. J. Biol. Chem..

[62] Hänggi M., Bannwarth W., Stunnenberg H.G. (1986). Conserved TAAAT motif in vaccinia virus late promoters: Overlapping TATA box and site of transcription initiation. EMBO J..

[63] International Committee on Taxonomy of Viruses. http://www.ictvonline.org/taxonomyHistory.asp?taxnode_id=20151927&taxa_name=Asfarviridae.

[64] Sogo J.M., Almendral J.M., Talavera A., Vinuela E. (1984). Terminal and internal inverted repetitions in African swine fever virus DNA. Virology.

[65] Tulman E.R., Delhon G.A., Ku B.K., Rock D.L. (2009). African swine fever virus. Curr. Top. Microbiol. Immunol..

[66] Kuznar J., Salas M.L., Vinuela E. (1980). DNA-dependent RNA polymerase in African swine fever virus. Virology.

[67] Salas J., Salas M.L., Vinuela E. (1988). Effect of inhibitors of the host cell RNA polymerase II on African swine fever virus multiplication. Virology.

[68] Rodríguez J.M., Salas M.L. (2013). African swine fever virus transcription. Virus Res..

[69] Moss B. (1996). Genetically engineered poxviruses for recombinant gene expression, vaccination, and safety. Proc. Natl. Acad. Sci. USA.

[70] Rodríguez J.M., Salas M.L., Vinuela E. (1996). Intermediate class of mRNAs in African swine fever virus. J. Virol..

[71] Garcia-Escudero R., Vinuela E. (2000). Structure of African swine fever virus late promoters: Requirement of a TATA sequence at the initiation region. J. Virol..

[72] Yates P.R., Dixon L.K., Turner P.C. (1995). Promoter analysis of an African swine fever virus gene encoding a putative elongation factor. Biochem. Soc. Trans..

[73] Van Etten J.L., Graves M.V., Müller D.G., Boland W., Delaroque N. (2002). Phycodnaviridae—Large DNA algal viruses. Arch. Virol..

[74] Van Etten J.L., Meints R.H. (1999). Giant viruses infecting algae. Annu. Rev. Microbiol..

[75] Wilson W.H., van Etten J.L., Allen M.J. (2009). The *Phycodnaviridae*: The story of how tiny giants rule the world. Curr. Top. Microbiol. Immunol..

[76] International Committee on Taxonomy of Viruses. http://www.ictvonline.org/taxonomyHistory.asp?taxnode_id=20153552&taxa_name=Phycodnaviridae.

[77] Kang M., Graves M., Mehmel M., Moroni A., Gazzarrini S., Thiel G., Gurnon J.R., van Etten J.L. (2004). Genetic diversity in chlorella viruses flanking *kcv*, a gene that encodes a potassium ion channel protein. Virology.

[78] Kawasaki T., Tanaka M., Fujie M., Usami S., Yamada T. (2004). Immediate early genes expressed in chlorovirus infections. Virology.

[79] Chuchird N., Nishida K., Kawasaki T., Fujie M., Usami S., Yamada T. (2002). A variable region on the chlorovirus CVK2 genome contains five copies of the gene for Vp260, a viral-surface glycoprotein. Virology.

[80] Yamada T., Hiramatsu S., Songsri P., Fujie M. (1997). Alternative expression of a chitosanase gene produces two different proteins in cells infected with Chlorella virus CVK2. Virology.

[81] Graves M.V., Meints R.H. (1992). Characterization of the major capsid protein and cloning of its gene from algal virus PBCV-1. Virology.

[82] Sugimoto I., Hiramatsu S., Murakami D., Fujie M., Usami S., Yamada T. (2000). Algal-lytic activities encoded by Chlorella virus CVK2. Virology.

[83] Fitzgerald L.A., Graves M.V., Li X., Feldblyum T., Hartigan J., van Etten J.L. (2007). Sequence and annotation of the 314-kb MT325 and the 321-kb FR483 viruses that infect Chlorella Pbi. Virology.

[84] Fitzgerald L.A., Graves M.V., Li X., Feldblyum T., Nierman W.C., van Etten J.L. (2007). Sequence and annotation of the 369-kb NY-2A and the 345-kb AR158 viruses that infect Chlorella NC64A. Virology.

[85] Fitzgerald L.A., Boucher P.T., Yanai-Balser G.M., Suhre K., Graves M.V., van Etten J.L. (2008). Putative gene promoter sequences in the chlorella viruses. Virology.

[86] Van Etten J.L. (2003). Unusual life style of giant chlorella viruses. Ann. Rev. Genet..

[87] Wilson W.H., Schroeder D.C., Allen M.J., Holden M.T., Parkhill J., Barrell B.G., Churcher C., Hamlin N., Mungall K., Norbertczak H. (2005). Complete genome sequence and lytic phase transcription profile of a Coccolithovirus. Science.

[88] International Committee on Taxonomy of Viruses. http://www.ictvonline.org/taxonomyHistory.asp.taxnode_id=20153003&taxa_name=Iridoviridae.

[89] Darai G., Delius H., Clarke J., Apfel H., Schnitzler P., Flugel R.M. (1985). Molecular cloning and physical mapping of the genome of fish lymphocystis disease virus. Virology.

[90] Williams T., Barbosa-Solomieu V., Chinchar V.G. (2005). A decade of advances in Iridovirus research. Adv. Virus Res..

[91] Jakob N.J., Muller K., Bahr U., Darai G. (2001). Analysis of the first complete DNA sequence of an invertebrate iridovirus: Coding strategy of the genome of Chilo iridescent virus. Virology.

[92] Chinchar V.G., Yu K.H., Jancovich J.K. (2011). The molecular biology of frog virus 3 and other iridoviruses infecting cold-blooded vertebrates. Viruses.

[93] Barray S., Devauchelle G. (1987). Protein synthesis in cells infected by Chilo iridescent virus: Evidence for temporal control of three classes of induced polypeptides. Virology.

[94] D’Costa S.M., Yao H., Bilimoria S.L. (2001). Transcription and temporal cascade in Chilo iridescent virus infected cells. Arch. Virol..

[95] D’Costa S.M., Yao H.J., Bilimoria S.L. (2004). Transcriptional mapping in Chilo iridescent virus infections. Arch. Virol..

[96] Willis D.B. (1987). DNA sequences required for trans activation of an immediate- early frog virus 3 gene. Virology.

[97] Beckman W., Tham T.N., Aubertin A.M., Willis D.B. (1988). Structure and regulation of the immediate-early frog virus 3 gene that encodes ICR489. J. Virol..

[98] Willis D.B., Granoff A. (1985). Transactivation of an immediateearly frog virus 3 promoter by a virion protein. J. Virol..

[99] Pallister J., Goldie S., Coupar B., Hyatt A. (2005). Promoter activity in the 59 flanking regions of the Bohle iridovirus ICP 18, ICP 46 and major capsid protein genes. Arch. Virol..

[100] Nalcacioglu R., Ince I.A., Vlak J.M., Demirbag Z., van Oers M.M. (2007). The Chilo iridescent virus DNA polymerase promoter contains an essential AAAAT motif. J. Gen. Virol..

[101] Nalcacioglu R., Demirbag Z., Vlak J.M., van Oers M.M. (2003). Promoter analysis of the Chilo iridescent virus DNA polymerase and major capsid protein genes. Virology.

[102] Dizman Y.A., Demirbag Z., Ince I.A., Nalcacioglu R. (2012). Transcriptomic analysis of Chilo iridescent virus immediate early promoter. Virus Res..

[103] Goorha R. (1981). Frog virus 3 requires RNA polymerase II for its replication. J. Virol..

[104] Goorha R., Willis D.B., Granoff A. (1977). Macromolecular synthesis in cells infected by frog virus 3. VI. Frog virus 3 replication is dependent on the cell nucleus. J. Virol..

[105] Goorha R., Murti G., Granoff A., Tirey R. (1978). Macromolecular synthesis in cells infected by frog virus 3. VIII. The nucleus is a site of frog virus 3 DNA and RNA synthesis. Virology.

[106] Baroudy B.M., Moss B. (1982). Sequence homologies of diverse length tandem repetitions near ends of vaccinia virus genome suggest unequal crossing over. Nucleic Acids Res..

[107] Tidona C.A., Darai G. (1997). Molecular anatomy of lymphocystis disease virus. Arch. Virol. Suppl..

[108] Federici B.A., Bigot Y., Granados R.R., Hamm J.J., Miller L.K., Newton I., Stasiak K., Vlak J.M., Fauquet C.M., Mayo M.A., Maniloff J., Desselberger U., Ball L.A. (2005). Family Ascoviridae. Virus Taxonomy: 8th Report of the International Committee on Taxonomy of Viruses.

[109] International Committee on Taxonomy of Viruses. http://www.ictvonline.org/taxonomyHistory.asp?taxnode_id=20151919&taxa_name=Ascoviridae.

[110] Cheng X.W., Wang L., Carner G.R., Arif B.M. (2005). Characterization of three ascovirus isolates from cotton insects. J. Invertebr. Pathol..

[111] Asgari S., Davis J., Wood D., Wilson P., McGrath A. (2007). Sequence and organization of the Heliothis virescens ascovirus genome. J. Gen. Virol..

[112] Bigot Y., Rabouille A., Sizaret P.Y., Hamelin M.H., Periquet G. (1997). Particle and genomic characterization of a new member of the Ascoviridae, *Diadromus pulchellus ascovirus*. J. Gen. Virol..

[113] Cheng X.W., Carner G.R., Arif B.M. (2000). A new ascovirus from *Spodoptera exigua* and its relatedness to the isolate from *Spodoptera frugiperda*. J. Gen. Virol..

[114] Salem T.Z., Turney C.M., Wang L., Xue J., Wan X.-F., Cheng X.-W. (2008). Transcriptional analysis of a major capsid protein gene from *Spodoptera exigua ascovirus 5a*. Arch. Virol..

[115] Zhao K., Cui L.W. (2003). Molecular characterization of the major virion protein gene from the *Trichoplusia ni ascovirus*. Virus Genes.

[116] Stasiak K., Renault S., Demattei M.V., Bigot Y., Federici B.A. (2003). Evidence for the evolution of ascoviruses from iridoviruses. J. Gen. Virol..

[117] Chinchar V.G., Hyatt A., Miyazaki T., Williams T. (2009). Family *Iridoviridae*: Poor viral relations no longer. Curr. Top. Microbiol. Immunol..

[118] Piégu B., Asgari S., Bideshi D., Federici B.A., Bigot Y. (2015). Evolutionary relationships of iridoviruses and divergence of ascoviruses from invertebrate iridoviruses in the superfamily Megavirales. Mol. Phylogenet. Evol..

[119] Zauberman N., Mutsafi Y., Halevy D.B., Shimoni E., Klein E., Xiao C., Sun S., Minsky A. (2008). Distinct DNA exit and packaging portals in the virus *Acanthamoeba polyphaga mimivirus*. PLoS Biol..

[120] Xiao C., Kuznetsov Y.G., Sun S., Hafenstein S.L., Kostyuchenko V.A., Chipman P.R., Suzan-Monti M., Raoult D., McPherson A., Rossmann M.G. (2009). Structural studies of the giant mimivirus. PLoS Biol..

[121] Rodrigues R.A., dos Santos Silva L.K., Dornas F.P., de Oliveira D.B., Magalhães T.F., Santos D.A., Costa A.O., de Macêdo Farias L., Magalhães P.P., Bonjardim C.A. (2015). Mimivirus fibrils are important for viral attachment to the microbial world by a diverse glycoside interaction repertoire. J. Virol..

[122] Legendre M., Santini S., Rico A., Abergel C., Claverie J.M. (2011). Breaking the 1000-gene barrier for Mimivirus using ultra-deep genome and transcriptome sequencing. Virol. J..

[123] Fischer M.G., Allen M.J., Wilson W.H., Suttle C.A. (2010). Giant virus with a remarkable complement of genes infects marine zooplankton. Proc. Natl. Acad. Sci. USA.

[124] Colson P., de Lamballerie X., Yutin N., Asgari S., Bigot Y., Bideshi D.K., Cheng X.W., Federici B.A., van Etten J.L., Koonin E.V. (2013). “Megavirales”, a proposed new order for eukaryotic nucleocytoplasmic large DNA viruses. Arch. Virol..

[125] Reteno D.G., Benamar S., Khalil J.B., Andreani J., Armstrong N., Klose T., Rossmann M., Colson P., Raoult D., La Scola B. (2015). Faustovirus, an asfarvirus-related new lineage of giant viruses infecting amoebae. J. Virol..

[126] Philippe N., Legendre M., Doutre G., Couté Y., Poirot O., Lescot M., Arslan D., Seltzer V., Bertaux L., Bruley C. (2013). Pandoraviruses: Amoeba viruses with genomes up to 2.5 Mb reaching that of parasitic eukaryotes. Science.

[127] Legendre M., Bartoli J., Shmakova L., Jeudy S., Labadie K., Adrait A., Lescot M., Poirot O., Bertaux L., Bruley C. (2014). Thirty-thousand-year-old distant relative of giant icosahedral DNA viruses with a pandoravirus morphology. Proc. Natl. Acad. Sci. USA.

[128] Levasseur A., Andreani J., Delerce J., Bou Khalil J., Robert C., La Scola B., Raoult D. (2016). Comparison of a modern and fossil pithovirus reveals its genetic conservation and evolution. Genome Biol. Evol..

[129] Legendre M., Lartigue A., Bertaux L., Jeudy S., Bartoli J., Lescot M., Alempic J.M., Ramus C., Bruley C., Labadie K. (2015). In-depth study of *Mollivirus sibericum*, a new 30,000-y-old giant virus infecting Acanthamoeba. Proc. Natl. Acad. Sci. USA.

[130] McLysaght A., Baldi P.F., Gaut B.S. (2003). Extensive gene gain associated with adaptive evolution of poxviruses. Proc. Natl. Acad. Sci. USA.

[131] Hughes A.L., Friedman R. (2005). Poxvirus genome evolution by gene gain and loss. Mol. Phylogenet. Evol..

[132] Elde N.C., Child S.J., Eickbush M.T., Kitzman J.O., Rogers K.S., Shendure J., Geballe A.P., Malik H.S. (2012). Poxviruses deploy genomic accordions to adapt rapidly against host antiviral defenses. Cell.

[133] Dixon L.K., Chapman D.A., Netherton C.L., Upton C. (2013). African swine fever virus replication and genomics. Virus Res..

[134] Portugal R., Coelho J., Höper D., Little N.S., Smithson C., Upton C., Martins C., Leitão A., Keil G.M. (2015). Related strains of African swine fever virus with different virulence: Genome comparison and analysis. J. Gen. Virol..

[135] Huang Y., Huang X., Liu H., Gong J., Ouyang Z., Cui H., Cao J., Zhao Y., Wang X., Jiang Y. (2009). Complete sequence determination of a novel reptile iridovirus isolated from soft-shelled turtle and evolutionary analysis of Iridoviridae. BMC Genomics.

[136] Koonin E.V., Krupovic M., Yutin N. (2015). Evolution of double-stranded DNA viruses of eukaryotes: From bacteriophages to transposons to giant viruses. Ann. N. Y. Acad. Sci..

[137] Nasir A., Kim K.M., Caetano-Anolles G. (2012). Giant viruses coexisted with the cellular ancestors and represent a distinct supergroup along with superkingdoms Archaea, Bacteria and Eukarya. BMC Evol. Biol..

[138] Moss B. (2013). Reflections on the early development of poxvirus vectors 2013. Vaccine.

